# Redox Mechanisms of Silica-Supported Ni Particles: An X-Ray Absorption Fine Structure Investigation

**DOI:** 10.3390/ma19081509

**Published:** 2026-04-09

**Authors:** Eka Novitasari, Kodai Ohta, Asaka Azuma, Yasuhiro Niwa, Masao Kimura, Yasuhiro Inada

**Affiliations:** 1 Graduate School of Life Sciences, Ritsumeikan University, 1-1-1 Noji-Higashi, Kusatsu 525-8577, Japan; 2 Photon Factory, High Energy Accelerator Research Organization, 1-1 Oho, Tsukuba 305-0801, Japan; yasuhiro.niwa@kek.jp (Y.N.); masao.kimura@kek.jp (M.K.)

**Keywords:** redox reaction, in situ XAFS, silica-supported Ni particles

## Abstract

The redox mechanisms of silica-supported Ni particles were investigated using their in situ X-ray absorption fine structure, providing mechanistic insights into partially reduced NiO and partially oxidized metallic Ni. The results of surface oxidation of partially reduced NiO particles at room temperature revealed that the surface was not fully covered with metallic Ni and that metallic Ni had also formed within the particle interior. During NiO particle reduction, the process initiates at specific surface sites, and before the metallic Ni phase fully covers the surface, O^2−^ ions are expelled from the particle. Conversely, the oxidation of metallic Ni particles progresses inward from the surface, with an accompanying increase in the thickness of the NiO layer that forms upon O_2_ exposure at room temperature. This mechanism is supported by observations that the reduction of a thin NiO shell on metallic Ni particles was completed below 200 °C, while reduction temperatures shifted to higher values as the NiO layer thickness increased. The distinct oxidation and reduction mechanisms are attributed to differences in the migration direction of O^2−^ ions. During reduction, it is proposed that O^2−^ ions within the particles migrate to the surface along the interface between the NiO phase and the metallic Ni phase. This study elucidates the detailed mechanism behind the redox interconversion between NiO and metallic Ni in solid catalyst particles.

## 1. Introduction

Extensive research has highlighted the importance of redox reactions involving solid Ni particles in various applications. For example, in solid oxide fuel cells, the oxidation of the Ni catalyst under non-reducing gas environments can cause degradation and worsen device performance [1,2]. The chemical state of supported Ni catalysts continuously changes during the conversion of biomass and other resources to syngas via chemical looping gasification [3,4,5] and combustion [6,7,8,9]. Therefore, understanding the kinetics and mechanisms behind the oxidation and reduction of supported Ni catalysts is crucial, as it will enable better control over catalytic activity, stability, and regeneration cycles.

Various studies have investigated the reduction and oxidation behavior of Ni species using different techniques. Richardson et al. [10] investigated the reduction process of bulk NiO using X-ray diffraction (XRD), observing simultaneous NiO loss and Ni appearance at 175 °C. Their results revealed three consecutive kinetic stages: an initial induction period associated with Ni nucleation, an acceleration period attributed to the autocatalytic growth of Ni clusters, and a subsequent pseudo-first-order reaction period. At the later stage of the reduction, the accumulation of adsorbed water molecules inhibited H_2_ access and H_2_O removal, indicating a change in the reaction mechanism. The kinetic behavior of metallic Ni formation and NiO disappearance shown in that study was found to be inconsistent with the classical shrinking-core model, in which the Ni–NiO interface moves toward the center of the crystal grain, leaving a porous metallic product layer to enable H_2_ insertion and H_2_O removal [10].

Sá et al. [11] studied the reduction of NiO by applying time-resolved resonant inelastic X-ray scattering (RIXS) spectroscopy to monitor the hydrogen reduction of NiO nanoparticles during temperature-programmed reduction (TPR). In their study, in situ RIXS maps were collected during heating in H_2_/He, enabling the simultaneous monitoring of unoccupied electronic states (via X-ray absorption spectroscopy, XAS) and occupied electronic states. The XAS analysis showed a low-energy shift in the ionization threshold, corresponding to the valence change from Ni^2+^ to Ni^0^. The reduction reaction proceeded in a single step in the temperature range of approximately 230 °C to 320 °C. Complementary quadrupole mass spectrometry measurements detected water formation, supporting the XAS results.

Jeangros et al. [12] studied the oxidation mechanism of Ni particles using a transmission electron microscope (TEM). An in situ environmental TEM combined with an electron energy-loss spectroscopy (EELS) methodology, integrating TEM imaging, diffraction, and temperature-resolved EELS under a controlled gas atmosphere [13], enabled the correlation of local structural changes with the evolution of Ni/NiO chemical states. Their results suggest that a built-in field effect controls the initial oxidation stage, followed by the migration of Ni^2+^ ions along NiO grain boundaries, leading to the formation of randomly orientated NiO crystallites and internal voids. Their work highlighted the important role of electric fields, although they noted that studies on electric field strength require further validation. While techniques such as electron holography may eventually address this challenge [14], complementary analysis, such as investigating the in situ X-ray absorption fine structure (XAFS), can provide valuable insights into the evolution of oxidation states, especially in supported Ni catalysts under intermediate redox conditions. In situ XAFS is a powerful tool for characterizing chemical states and is widely applied in the study of redox processes in supported metal species [15,16,17,18,19,20,21].

Recent in situ XAFS studies have provided significant insights into the redox interconversion between NiO and Ni [22,23,24]. In the most recent study [24], the chemical state evolution of Ni species supported on SiO_2_ was investigated as a function of the particle size. These studies revealed that interfacial NiO species stabilized by SiO_2_ resist NiO reduction. This behavior can be associated with the strong metal–support interaction (SMSI) effect [25,26,27], indicating that even SiO_2_, which is typically considered an inert support, can strongly interact with Ni under appropriate conditions [28]. Furthermore, it was shown that a Ni core–NiO shell state, where only the particle surface layer is oxidized, is initially formed during the oxidation process of metallic Ni particles. These findings highlight the importance of particle size control in optimizing redox properties in catalytic applications.

A previous in situ XAFS study [24] revealed that stabilized NiO remains at the interface with SiO_2_ during the reduction process of NiO on SiO_2_. However, it remains unclear how the NiO portion not located at the interface is reduced. In this study, we aimed to more precisely clarify the reduction mechanism of NiO particles by generating a partially reduced state of NiO and analyzing the proportion of metallic Ni present on the particle surface. Furthermore, previous research showed that the 5–9 Å region on the particle surface is first converted to NiO during the oxidation process of metallic Ni particles [24]. In this study, we investigated the mechanism behind the oxidation process of metallic Ni from the particle surface to the interior by performing in situ XAFS analysis of the TPR process in metallic Ni particles under varying degrees of partial oxidation. The purpose of this study was to conduct a detailed analysis of the reduction of non-interfacial NiO and the oxidation of the Ni core within the NiO shell, which have not been previously elucidated [24], and to explore the overall redox mechanism of SiO_2_-supported Ni particles.

To investigate these processes, in situ XAFS analysis was performed on SiO_2_-supported Ni particles with precisely controlled redox states. Through the interruption of the reduction of NiO particles, intermediate reduction states were intentionally generated. Analyzing the surface oxidation in these states allowed us to quantify the exposed metallic Ni both on the surface and within the particles. Systematically varying the extent of partial reduction provided insights into the reduction mechanism of NiO particles. Similarly, we conducted in situ XAFS analysis during the reduction (TPR) of partially oxidized metallic Ni particles, which were prepared by interrupting the oxidation of metallic Ni. This allowed us to investigate the progression of oxidation within the metallic Ni particles and elucidate the oxidation mechanism. This approach is particularly useful in understanding the chemical transformation of Ni catalyst particles during redox cycling, offering novel insights into the redox mechanism of SiO_2_-supported Ni species.

## 2. Experimental Procedure

### 2.1. Sample Preparation and Characterization

SiO_2_-supported Ni catalysts with a Ni loading of 9.4 wt% were prepared via the impregnation method, employing malonic acid in the precursor solution to control particle size. Ni(NO_3_)_2_•6H_2_O (FUJIFILM Wako Pure Chemicals, Osaka, Japan, 98% purity) was dissolved in deionized water to prepare a 34 mM aqueous Ni^2+^ solution. Malonic acid (FUJIFILM Wako Pure Chemicals) was added to this solution in an equimolar amount to that of Ni^2+^. A calculated amount of SiO_2_ (CARiACT Q-15; Fuji Sylisia Chemical, Kasugai, Japan; specific surface area: 192 m^2^/g) was suspended in the solution under continuous stirring for 1 h at 70 °C. The mixture was dried at 70 °C for 72 h and ground into a fine powder. The obtained powder was calcined at 600 °C in air for 3 h and then reduced under a dilute H_2_ flow (10 vol% H_2_ in Ar, total flow rate: 200 cm^3^/min) at 700 °C for 1 h. The reduced sample was treated at 600 °C for 1 h under a dilute O_2_ flow (10 vol% O_2_ in Ar, total flow rate: 200 cm^3^/min) to obtain NiO/SiO_2_. This single reduction–oxidation cycle was performed as previously reported [24,29,30].

The crystallinity of Ni/SiO_2_ was analyzed via XRD using an Ultima IV instrument (Rigaku, Tokyo, Japan) with Cu Kα radiation. The crystallite size was calculated using the Scherrer equation. The particle size of the Ni species was measured via TEM using a JEM-2100Plus instrument (JEOL, Tokyo, Japan). The Ni loading was determined via X-ray fluorescence (XRF) analysis using a Supermini fluorescent X-ray spectrometer (Rigaku, Tokyo, Japan).

### 2.2. In Situ XAFS Measurement

In situ XAFS measurements at the Ni K edge were conducted in transmission mode at the BL-9C beamline of the Photon Factory (High Energy Accelerator Research Organization, Tsukuba, Japan). Higher-order reflections were eliminated by detuning the monochromator crystals. The prepared sample was placed in a quartz flow-type cell. The XAFS spectra were processed using the Athena and Artemis software (Version 0.9.26) [31]. Details of the analysis are provided in Supplementary Materials.

The SiO_2_-supported NiO particles were first partially or completely reduced under a H_2_ atmosphere at various predetermined temperatures, as established in a previous study [24]. During partial reduction, the composition of NiO and Ni was monitored via linear combination fitting (LCF) analysis of the measured XANES spectra. The treatment temperature was adjusted accordingly to quench the sample state during the TPR process. The reduced sample was cooled to room temperature under continuous H_2_ flow. The gas was then switched to diluted O_2_ at room temperature to oxidize the particle surface. After O_2_ treatment, the flow gas was changed back to H_2_ (10 vol% balanced by He; total flow rate: 100 cm^3^/min), and in situ XAFS measurements were performed during the TPR process up to 700 °C (heating rate: 10 °C/min) to achieve the complete reduction of the remaining NiO species.

To achieve a partially oxidized state of metallic Ni, with the TPO process arrested midway, metallic Ni generated via TPR was treated in a diluted O_2_ flow (10 vol% balanced by He; total flow rate: 100 cm^3^/min) to promote partial oxidation up to various predetermined temperatures, based on previous findings [24]. The treatment temperature was adjusted by monitoring the composition of the produced NiO via LCF analysis of the measured XANES spectra. The TPR process of the partially oxidized Ni particles was analyzed via in situ XAFS measurements until complete reduction was achieved.

After the partial reduction of NiO or oxidation of metallic Ni, the sample was cooled to room temperature, and XANES measurements were continued for 10–15 min to confirm that no further spectral changes were observed. This state is regarded as a partially reduced or partially oxidized quenched state in this study.

## 3. Results and Discussion

### 3.1. Sample Characterization

**Figure 1:**
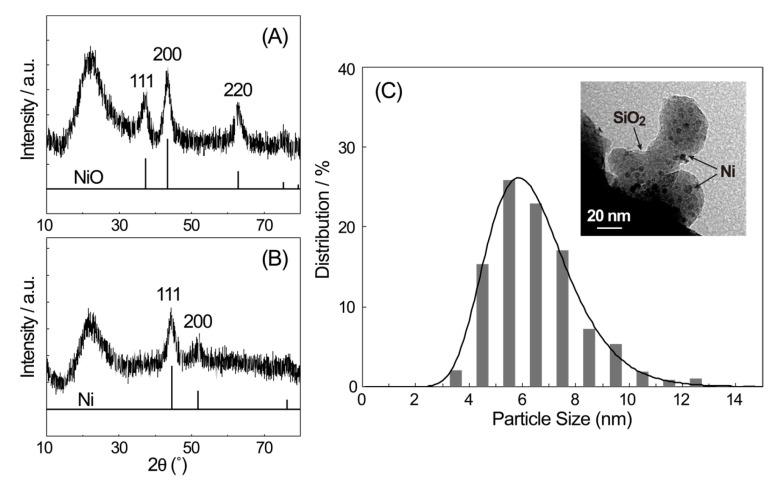
XRD patterns of SiO_2_-supported samples after the oxidation (**A**) and reduction (**B**) treatments and histogram of particle size distribution for the metallic Ni species, together with an example of the observed TEM images (**C**).

Figure 1 presents the XRD pattern of the as-synthesized SiO_2_-supported samples. A broad diffraction peak around 22° was assigned to amorphous SiO_2_; this peak remained observable following both the oxidation and reduction treatments. Distinct diffraction peaks at 37.60°, 43.44°, and 63.04° were assigned to the (111), (200), and (220) planes of NiO, respectively (PDF card No. 01-078-4373). This NiO is a non-stoichiometric compound and should be appropriately written as Ni_1−*x*_O. However, in this paper, it will be referred to as NiO to clearly show the difference from the reduction composition. Following reduction treatment, diffraction peaks appeared at 44.38° and 52.12°, which were assigned to the (111) and (200) planes of metallic Ni, respectively (PDF card No. 00-004-0850), confirming the formation of crystalline Ni particles. Crystallite sizes, estimated using the Scherrer equation for the (200) peak after correction for instrumental line broadening, were 6 nm for NiO and 5 nm for Ni. This observation (Ni being smaller than NiO) contrasts with previous reports [10,12] where metallic Ni crystallite sizes were typically 2 to 10 times larger than NiO before reduction. This difference is likely attributed to the presence of supporting materials in our system, where SMSI at the Ni-SiO_2_ interface [28] induces an anchoring effect that limits the mobility of Ni species, thereby suppressing particle aggregation and growth [32,33].

The particle size distribution of the reduced sample is shown in Figure 1, along with a representative TEM image. The light gray areas in the TEM image represent the amorphous SiO_2_, and the black dots on top of it are the supported Ni particles. The black area to the lower left is a part of the grid used for TEM observation. The particle size distribution was obtained by analyzing 580 particles from multiple TEM images. The diameters of the metallic Ni particles primarily ranged from 4 to 8 nm, with an average diameter of 6.2 ± 0.3 nm, calculated using a log-normal distribution function. This value agrees well with the crystallite size determined via XRD measurement. The particles are treated as approximately spherical, although some deviation in morphology is expected. The particle size of the present sample, prepared via impregnation with malonic acid, was consistent with values reported in a previous study using identical conditions [24]. This consistency is attributed to the enhanced dispersion of supported particles, resulting from the addition of organic materials [34,35,36,37]. The Ni loading was determined via XRF analysis to be 9.4 wt% (Supplementary Information, Figure S1).

### 3.2. Reduction Process of SiO_2_-Supported NiO Particles

The XANES spectral evolution during the TPR process of NiO supported on SiO_2_ is shown in Figure S2. The initial spectrum matched the NiO standard but evolved into that of metallic Ni during heating, exhibiting multiple isosbestic points. The presence of these points indicates that the conversion of NiO to Ni proceeds directly without stable intermediate phase formation. To further examine the possible formation of other phases, such as Ni(OH)_2_ and Ni_2_SiO_4_, the reference XANES spectra of these compounds were compared with the measured spectra in Figure S3. No spectral features characteristic of Ni(OH)_2_ or Ni_2_SiO_4_ were observed throughout the TPR process. These results suggest that, within the sensitivity of the present measurements, the reduction occurs directly between NiO and metallic Ni without detectable additional phases.

**Figure 2:**
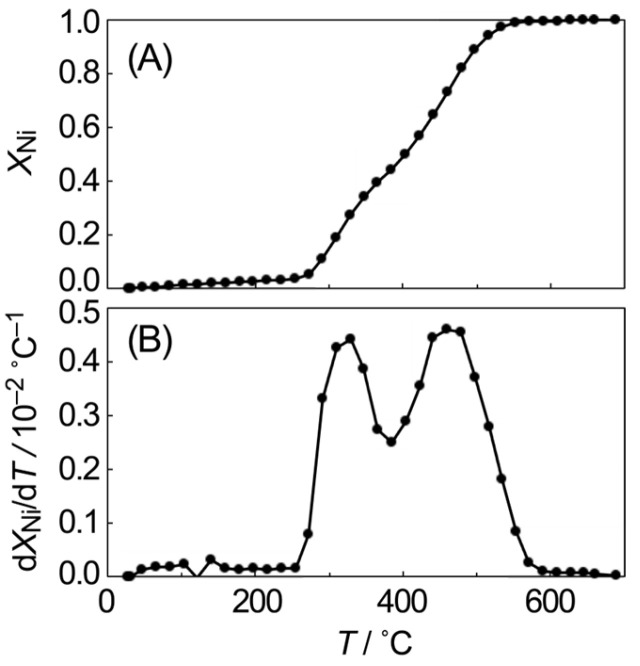
Change in the mole fraction of metallic Ni (**A**) and its first derivative (**B**) during the TPR process of NiO supported on SiO_2_ as a function of the temperature.

The mole fraction of metallic Ni (*X*_Ni_), determined via LCF analysis of the XANES spectra, is presented in Figure 2 as a function of the temperature (*T*), along with its first derivative (d*X*_Ni_/d*T*). The *X*_Ni_ value was affected by a slight temperature-dependent change in the XANES spectrum, which was estimated to be about 5%. The slight increase in *X*_Ni_ up to 250 °C represents the estimation error. The d*X*_Ni_/d*T* exhibited two distinct peaks at 330 °C and 470 °C, with a local minimum at 400 °C, suggesting the presence of two types of NiO with differing reduction properties. This behavior is consistent with a previous report [24] indicating that NiO remains at the SiO_2_ interface at intermediate temperatures around 400 °C. The NiO species stabilized at the SiO_2_ interface, which requires temperatures above 400 °C for reduction, is attributed to strong metal–support interaction [25,26,27].

**Figure 3:**
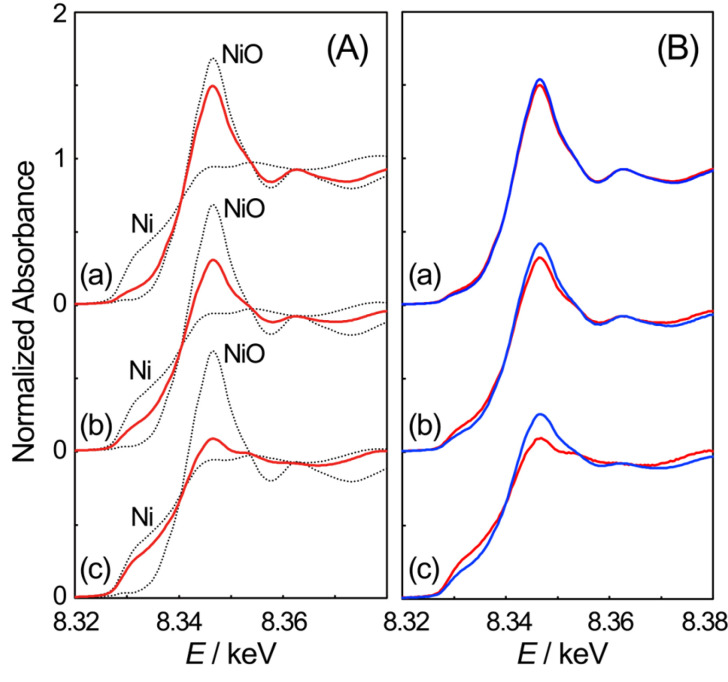
XANES spectra of NiO particles supported on SiO_2_ after the partial reduction (**A**) at 336 °C (a), 403 °C (b), and 455 °C (c) together with reference spectra shown as dotted lines. In (**B**), the XANES spectra are compared before (red lines) and after (blue lines) the O_2_ exposure at room temperature.

To gain deeper mechanistic insight into the H_2_ reduction of NiO particles supported on SiO_2_, a stepwise partial reduction was carried out at elevated temperatures. The heating process was interrupted at specific temperatures (336 °C, 403 °C, and 455 °C) to achieve the target *X*_Ni_ values. This procedure resulted in samples where the reduction of NiO particles was halted during the TPR process. The system was then cooled under a flow of H_2_ to preserve their reduced state. This procedure produced a series of partially reduced samples containing NiO and metallic Ni phases in varying ratios. Figure 3A shows the XANES spectra of the obtained samples measured at room temperature. The relative proportions of NiO and metallic Ni were determined via LCF analysis of the XANES spectra. For samples where the partial reduction was halted at 336 °C, 403 °C, and 455 °C, the *X*_Ni_ values were 0.25, 0.48, and 0.79, respectively. In addition, a sample with an *X*_Ni_ value of 1.00 was prepared via heating to 700 °C under H_2_ flow to ensure that NiO was completely reduced.

After the partial reduction treatment, each sample underwent a surface oxidation treatment at room temperature. Each sample was exposed to 10 vol% O_2_ diluted in He at a total flow rate of 100 cm^3^/min, without external heating. This step was designed to selectively oxidize the metallic Ni exposed on the particle surface, thereby differentiating surface metallic Ni from bulk metallic Ni. This approach revealed metallic Ni sites with varying accessibility for O_2_. Figure 3B illustrates the changes observed in the XANES spectra following O_2_ exposure at room temperature. Exposure to O_2_ increased the white line peak absorbance and shifted the absorption edge to higher energy. These changes correspond to the partial oxidation of metallic Ni to NiO, with previous studies demonstrating that this room temperature change indicates the oxidation of metallic Ni on the particle surface [22,23,24]. Figure 3B shows that metallic Ni species were present on the particle surface in all samples, despite variations in the overall amount of Ni produced through partial reduction.

The *X*_Ni_ values, determined via LCF calculation, are summarized in Table 1. Surface oxidation of the fully reduced sample (initial *X*_Ni_ = 1.00) revealed that 29% of the metallic Ni (corresponding to a decrease in *X*_Ni_ of 0.29, Table 1) was located at the particle surface, with the remaining 71% found in the interior. This value is taken as a reference for the maximum fraction of Ni located at the particle surface. Thus, if the surface layer of a NiO particle were completely covered with metallic Ni, O_2_ exposure would be expected to decrease *X*_Ni_ by 0.29. However, for all partially reduced samples shown in Table 1, the observed decrease in *X*_Ni_ was significantly less than 0.29. This discrepancy indicates that not all reduced Ni species are equally accessible for O_2_, meaning that the reduced Ni species are not confined to the particle surface but are distributed within the particle interior, where access to gaseous O_2_ is impossible.

For instance, in the sample partially reduced to an initial *X*_Ni_ of 0.48, if its particle surface layer had been completely reduced, *X*_Ni_ would have been expected to decrease to 0.19 (0.48–0.29) upon O_2_ exposure at room temperature. However, the observed *X*_Ni_ after oxidation was 0.37, representing a decrease of only 0.11 (Table 1). This implies that of the 48% reduced Ni species, only 11% (the oxidized fraction) was located on the particle surface, while the remaining 37% (0.48–0.11) was situated inside the particle. Similarly, for the sample with an initial *X*_Ni_ of 0.25, only 5% of the reduced metallic Ni (a decrease of 0.05, Table 1) was present in the particle surface layer, with the remaining 20% (0.25–0.05) located internally. Furthermore, even when reduction was continued to *X*_Ni_ = 0.79, the observed decrease in *X*_Ni_ due to surface oxidation was 0.24 (Table 1), which is less than the 0.29 expected for a fully reduced surface. This indicates that even at high reduction levels, the entire particle surface was not uniformly reduced, and some NiO remained on portions of the particle surface.

**Figure 4:**
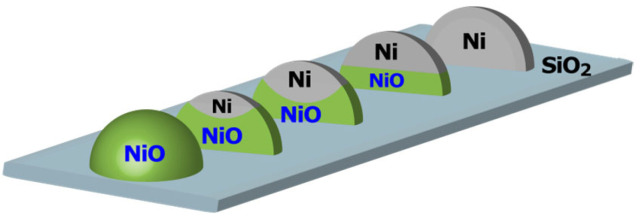
Schematic of the reduction process of NiO particles.

Collectively, this analysis demonstrates that partially reduced metallic Ni species are distributed throughout both the surface and interior of the particles. As schematically illustrated in Figure 4, the reduction of NiO particles is thus believed to initiate at localized sites on the particle surface and progressively advance toward the particle interior. To further clarify the validity of the proposed mechanism, we compared it with the conventional shrinking-core model, which is often applied to solid–gas reduction processes. In this model, reduction proceeds via the formation of a porous product layer, allowing gas diffusion and resulting in a well-defined reaction front moving from the surface toward the core [38]. If such a mechanism were operative in the present system, the reduced Ni phase would exhibit a highly porous structure with a large accessible metallic surface area. Consequently, upon exposure to O_2_ at room temperature, a significantly larger fraction of Ni would be expected to undergo oxidation. However, only a limited fraction of Ni is oxidized under these conditions, indicating that the shrinking-core model is not applicable.

This reduction mechanism contrasts sharply with the oxidation process of metallic Ni particles, where the particle surface is initially covered by a NiO layer. This contrasting behavior is likely due to the difficulty with which O^2−^ ions dissociate from the remaining NiO phase within the particle interior once the NiO particle surface begins to be covered with a metallic Ni layer during reduction. It is hypothesized that metallic Ni grains form at the particle surface, facilitating the expulsion of oxide ions from the particle interior across the interface with the NiO phase. It should be noted that this interpretation is based on changes in the average Ni oxidation state derived from XANES and assumes a simplified particle model. As XANES provides ensemble-averaged information, the spatial distribution of reduced species cannot be directly resolved.

**Figure 5:**
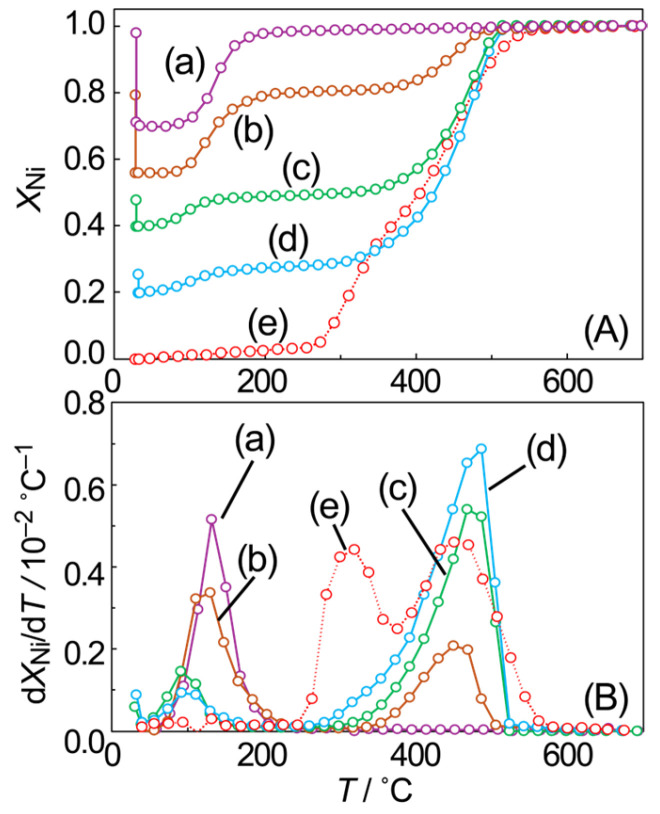
Temperature dependence of the mole fraction of Ni (*X*_Ni_) (**A**) and its first derivative (**B**) during the TPR process of NiO/SiO_2_. Data are presented for previously reduced NiO particles, subsequently exposed to O_2_ at room temperature, with initial *X*_Ni_ values of 1.00 (a), 0.79 (b), 0.48 (c), and 0.25 (d). For comparison, the TPR process of NiO particles (e) without prior reduction treatment is also shown.

After the surface oxidation of the partially reduced NiO particles, in situ XAFS measurements were performed during the subsequent TPR process up to 700 °C under H_2_ flow. Both residual NiO from the partial reduction and NiO formed on the particle surface through O_2_ exposure at room temperature were present. Consequently, the reduction of these NiO particles was expected to proceed in multiple stages. Figure 5 shows the temperature dependence of *X*_Ni_ and its first derivative curve, obtained from in situ XAFS analysis of the TPR process. The rapid decrease in *X*_Ni_ at room temperature corresponds to the oxidation of metallic Ni components exposed on the particle surface upon O_2_ exposure. A portion of the NiO was reduced in the temperature range up to 200 °C, and the corresponding increase in *X*_Ni_ was consistent in magnitude with the decrease observed at room temperature. This suggests that unstable NiO is present on the particle surface, undergoing reduction below 200 °C. The first derivative curve indicated that the reduction of surface NiO peaked at approximately 110 °C, irrespective of the extent of initial partial reduction. This low-temperature reduction characteristic was attributed to the lattice mismatch caused by the presence of an internal metallic Ni core, as previously reported [24]. After the reduction of the surface NiO, the residual NiO was reduced at approximately 450 °C. This temperature corresponds to the higher-temperature stage of the two-stage reduction observed in the TPR process of NiO particles without prior reduction treatment (curve e in Figure 5. Previous studies [24] have attributed this to the reduction of NiO interacting with the SiO_2_ support. Therefore, it is reasonable to assume that the second stage of reduction of partially reduced NiO particles proceeds at approximately 450 °C. A schematic diagram illustrating the evolution of the intraparticle chemical state during this TPR process is shown in Figure S4, exemplified by the results for *X*_Ni_ = 0.48.

### 3.3. Oxidation Process of SiO_2_-Supported Ni Particles

Unlike the analysis of partially reduced NiO particles described in the previous section, this study involved in situ XAFS analysis of the TPR process for metallic Ni particles partially oxidized to varying degrees. We controlled the proportion of the NiO component by heating metallic Ni particles to various temperatures under a He-diluted O_2_ gas flow. The heating process was stopped at 119 °C, 191 °C, 236 °C, and 500 °C, yielding NiO compositions of 43.9%, 60.3%, 80.7%, and 100% respectively. The XANES spectra measured for these partial oxidation treatments are shown in Figure S5. It is also known that when metallic Ni particles are exposed to a diluted O_2_ atmosphere at room temperature (25 °C), 28.4% NiO is found on their surface [24]. We have demonstrated that the oxidation of the particle surface occurs at room temperature by examining the change in the oxidation ratio as a function of the particle size. This observation is consistent with the Cabrera and Mott model [39], which posits that oxidation is mediated by the contact potential difference between Ni metal and adsorbed O_2_.

**Figure 6:**
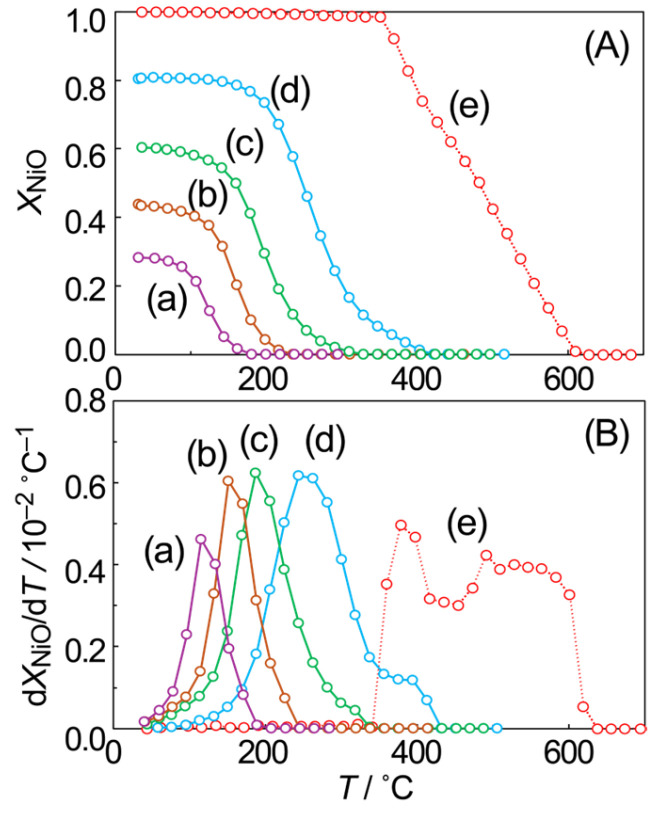
Temperature-dependent mole fraction of NiO (**A**) and its first derivative function (**B**) during the TPR process for partially oxidized Ni particles with initial NiO mole fractions of 0.28 (a), 0.44 (b), 0.60 (c), 0.81 (d), and 1.00 (e).

Figure 6 illustrates the temperature-dependent mole fraction of NiO (*X*_NiO_) and its first derivative (d*X*_NiO_/d*T*), as determined via in situ XAFS analysis during TPR processes for Ni particles with varying initial NiO proportions. When NiO is present solely on the surface of metallic Ni particles (Figure 6 (a)), its reduction occurs at low temperatures, below 200 °C, consistent with previous reports [24]. Our study revealed that the onset temperature for NiO reduction increases with an increasing degree of partial oxidation. Furthermore, for initial *X*_NiO_ ≥ 0.6, the reduction reaction proceeds in two steps, demonstrated by a high-temperature shoulder in the first derivative curve. This two-step reduction mirrors that observed for bulk NiO particles, which also proceeds in two distinct temperature ranges (approximately 400 °C and 550 °C; see Figure 6 (e)). The components reduced at high temperatures are attributed to NiO species stabilized via SMSI with SiO_2_ [24], indicating the presence of such stabilized NiO. A similar phenomenon is observed in partially oxidized samples, appearing as a high-temperature shoulder in the first derivative curve of *X*_NiO_, as shown in Figure S6; this schematic diagram demonstrates that, when metallic Ni particles are oxidized from the surface, there must be a portion in contact with the SiO_2_ support. The NiO present there can be reasonably expected to be stabilized via SMSI.

The fraction of NiO reduced in the high-temperature region was estimated based on the change in normalized *X*_NiO_ over the corresponding temperature range, as shown in Figure S7. For the sample with *X*_NiO_ of 0.81, the component reduced above 300 °C is attributed to this stabilized NiO, accounting for approximately 25% of the initial NiO at room temperature. Similarly, approximately 16% of NiO is estimated to be located at the interface with SiO_2_ for *X*_NiO_ = 0.60. With the average 6.2 nm sized NiO particles used in this study, 65% of the NiO component required high temperatures for reduction due to stabilization at the SiO_2_ interface (see Figure S7). The proportion of this stabilized component in partially oxidized metallic Ni particles was lower than this value, which suggests that metallic Ni remains at the SiO_2_ interface even after partial oxidation. These findings indicate that the oxidation of metallic Ni particles proceeds inward from the surface, forming a metallic Ni core within the particle. As for components that require high temperatures for reduction, it is possible that they originate from thicker or more crystalline NiO domains formed when heated in O_2_. However, there should be parts of the surface of the metallic Ni particles in contact with the SiO_2_ support, and when these parts are oxidized to NiO, stabilization by the support should occur.

The TPR profiles of the NiO phase in partially oxidized Ni particles suggest a single, well-defined reduction process, apart from the high-temperature tailing discussed above (see Figure 6 (a–d)). This observation is inconsistent with a heterogeneous oxidation pathway that yields NiO phases of varying thicknesses at different locations within the particle [12]. Such an inconsistency arises because NiO layers of different thicknesses, particularly those in contact with either metallic Ni or bulk NiO, exhibit distinct reduction temperatures. For instance, surface NiO on metallic Ni reduces below 200 °C, whereas NiO layers with underlying bulk NiO require higher temperatures, as shown by the reduction of bulk NiO particles occurring only above 400 °C. The sigmoidal kinetics of the reduction process support the formation of a NiO layer of nearly uniform thickness on the metallic Ni core. Furthermore, this NiO layer is also expected to be in contact with the SiO_2_ support at the outer edge of the particle. The NiO components stabilized via this interaction are believed to be responsible for the observed higher-temperature reduction (the tailing).

**Figure 7:**
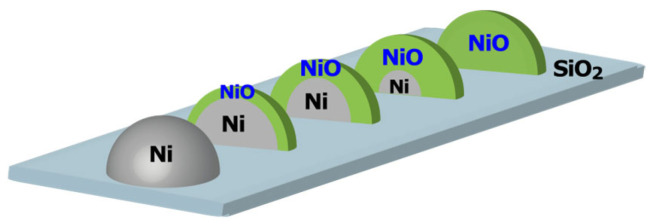
Schematic of the oxidation process of Ni particles.

Therefore, the most reasonable oxidation mechanism involves the formation of a NiO shell on a metallic Ni core, with the NiO layer thickness increasing as oxidation progresses (Figure 7). Railsback et al. [40] similarly reported that Ni particle oxidation proceeds via NiO shell formation, leading to internal cavity formation, as observed via TEM. While no evidence for the formation of such hollow NiO particles was obtained in this study, the progression of oxidation at the Ni/NiO interface, resulting in a shrinking metallic Ni core, is consistent with the oxidation model derived here.

Considering this oxidation model, the initial NiO shell thickness (*d*) can be estimated based on the *X*_NiO_ value at room temperature and the average particle size. The calculated *d* values are summarized in Table 2. To evaluate the reduction onset temperature for each NiO shell thickness, all initial *X*_NiO_ values were normalized to 1.0 (Figure S7), and *T*_red_ was defined as the temperature at which the normalized *X*_NiO_ value decreased to 0.95. The determined *T*_red_ values are also included in Table 2. It was found that *T*_red_ was low for thin NiO layers and increased with increasing NiO layer thickness. When the NiO layer is thick, the NiO further from the metallic Ni interface exhibits higher reduction resistance than NiO in direct contact with metallic Ni. The NiO formed when only the particle surface is oxidized (*X*_NiO_ = 0.28) represents the most unstable NiO, likely due to its poor interaction with the underlying metallic Ni core. Flege et al. reported that only a thin, metastable (111) NiO layer was formed below 200 °C, whereas the more thermodynamically stable (100) NiO was formed at higher temperatures [41]. This finding explains the observed increase in reduction temperature as the NiO layer becomes thicker. The systematic increase in *T*_red_ with increasing *X*_NiO_ suggests the formation of thicker (100) NiO layers, thereby supporting the proposed oxidation mechanism for metallic Ni particles shown in Figure 7.

Figure S8 shows the temperature-dependent change in *X*_NiO_ during the TPO process, where metallic Ni particles (average size 6.2 nm) supported on SiO_2_ were directly oxidized. The 31% NiO formed at room temperature upon O_2_ introduction represents a surface NiO layer. This proportion is consistent with a previous report [24] for metallic Ni particles of similar average size (6.7 nm). *X*_NiO_ remained almost constant up to ca. 80 °C, beyond which the oxidation reaction proceeded, permitting the migration of O^2−^ ions within the particles. This temperature corresponds to the *T*_red_ value (80 °C) observed for a NiO layer of corresponding thickness (*X*_NiO_ = 0.28). These results are consistent with the Cabrera-Mott model [39] and support a mechanism where Ni particle oxidation initiates at the surface and propagates inward by overcoming a kinetic barrier.

## 4. Conclusions

In this study, we performed in situ XAFS analysis during the TPR processes of partially reduced NiO particles and partially oxidized metallic Ni particles, thereby elucidating the redox mechanism of SiO_2_-supported Ni catalysts. The reduction of NiO particles did not proceed via a simple outside–in pathway. Instead, metallic Ni domains formed at the surface of NiO particles during the initial reduction stage and then spread into the adjacent NiO phases. This stepwise progression explains the multi-step reduction profile observed during TPR following the surface oxidation of partially reduced NiO. Consequently, metallic Ni and NiO phases coexist on the particle surface throughout the reduction process, maintaining a domain boundary that facilitates the expulsion of O^2−^ ions from the particle interior. On the other hand, the oxidation of metallic Ni particles forms a NiO shell around a Ni core, with the NiO shell thickness increasing as oxidation progresses. Experimental evidence supported this, showing that the reduction of the NiO phase occurred at higher temperatures when a thicker NiO shell was formed through more extensive oxidation of Ni. Thus, the reduction of NiO and the oxidation of metallic Ni proceed via distinct reaction pathways; i.e., NiO reduction is initiated at preferential surface sites, while Ni oxidation produces a uniform NiO layer. The irreversible redox mechanism identified in this study is interpreted as reflecting differences in the direction of O^2−^ ion migration. In both redox processes, the reaction is dominated by the movement of O^2−^ ions at the NiO/Ni interface. In the case of oxidation, the interface may exist within the particle, whereas in the case of reduction, the interface must be exposed, on the particle surface. This difference is interpreted as being the origin of the observed irreversibility.

## Figures and Tables

**Table 1:** The mole fraction of metallic Ni, *X*_Ni_, before and after O_2_ exposure at room temperature.

Before	After	Change with O_2_ Exposure
0.25	0.20	0.05
0.48	0.39	0.11
0.79	0.55	0.24
1.00	0.71	0.29

**Table 2:** NiO shell thickness and reduction onset temperature.

*X*_NiO_	*d*/Å	*T*_red_/°C
0.28	4	70
0.44	7	87
0.60	10	116
0.81	17	178
1.00	31 *	358

* Half the average particle size (6.2 nm).

## Data Availability

The original contributions presented in this study are included in the article/Supplementary Materials. Further inquiries can be directed to the corresponding author.

## Supplementary Materials

The following supporting information can be downloaded at https://www.mdpi.com/article/10.3390/ma19081509/s1, Figure S1: Calibration curve and measurement result of prepared sample in XRF analysis to analyze Ni loading; Figure S2: XANES spectral change during the TPR process for NiO particle supported on SiO_2_ from room temperature to 700 °C under a diluted H_2_ gas flow; Figure S3: The XANES spectra (black line) during the TPR process of SiO_2_-supported NiO particles were compared with those of NiO (red line), Ni(OH)_2_ (blue line), Ni_2_SiO_4_ (green line), and Ni foil (yellow line); Figure S4: A schematic diagram of the series of changes in the intraparticle chemical state during this TPR process. First, NiO particles supported on SiO2 were partially reduced via TPR until *X*_Ni_ = 0.48. This state was quenched and cooled to room temperature (a), and He-diluted O_2_ gas was introduced to oxidize the metallic Ni site exposed on the particle surface (b). The NiO formed on the particle surface (labeled as NiO*) has a metallic Ni core inside, and its environment is different from that of the other NiO located at the particle surface. Subsequently, in situ XAFS measurements were performed on the TPR process under a flow of He-diluted H_2_ gas, and it was found that reduction of an amount corresponding to NiO* had progressed at around 100 °C (c). Further heating led to the reduction of other NiO moieties at 450–500 °C, ultimately resulting in reduction to metallic Ni (d); Figure S5: The XANES spectra measured for the partial oxidation treatments; Figure S6: This diagram illustrates the behavior of the shoulder structure at high temperatures observed for the first derivative curve of *X*_NiO_ and the stabilization at the SiO_2_–NiO interface. The partial oxidation of Ni nanoparticles forms the NiO shell on the particle surface, and the reduction of the shell by H_2_ converts NiO to metallic Ni. However, because the NiO present at the interface between the particle and the support is likely stabilized via SMSI, it is expected that higher temperatures will be required for its reduction; Figure S7: The data in Figure 6A are plotted normalized to a value of 1.0 for *X*_NiO_ at room temperature. The actual value of *X*_NiO_ at room temperature is 0.28 (a), 0.44 (b), 0.60 (c), 0.81 (d), and 1.00 (e); Figure S8: The temperature change of *X*_NiO_ during the TPO process, in which the metallic Ni particles supported on SiO_2_ used in this study, with the average particle size of 6.2 nm, were directly oxidized. The 31% NiO produced at room temperature corresponds to the formation of a surface NiO layer upon exposure to O_2_ gas.
