# Supplementary material for: Redox Mechanisms of Silica-Supported Ni Particles: An X-Ray Absorption Fine Structure Investigation
Source: Materials (Basel). 2026 Apr 9;19(8):1509. doi: 10.3390/ma19081509 (PMC13117695; doi:10.3390/ma19081509)
Supplement: Supplementary file 1 [file materials-19-01509-s001.zip › materials-4217438-supplementary.pdf]

Supplementary Materials

Redox mechanisms of silica-supported Ni particles: an X-ray absorption fine structure investigation

Eka Novitasari, Kodai Ohta, Asaka Azuma, Yasuhiro Niwa, Masao Kimura, Yasuhiro Inada

XAFS Analysis

Analysis of the measured XAFS data was performed using the Demeter package [31]. The background absorbances in the pre- and post-edge region of the XAFS spectrum were calculated using the Athena code. The background absorbances were removed from the observed data to get the normalized XANES spectrum.

The linear combination fitting (LCF) analysis of the normalized XANES spectrum was conducted to determine the composition of the Ni species according to Eq. (1):

$$\mu_{\mathrm{o}}(E) = \sum_{i} a_i \mu_i(E), \quad (1)$$

where $\mu_{\mathrm{o}}(E)$ is the normalized XANES spectrum of the Ni catalysts and μ_i is the XANES spectrum of reference compound *i* with the mole fraction of a_i.

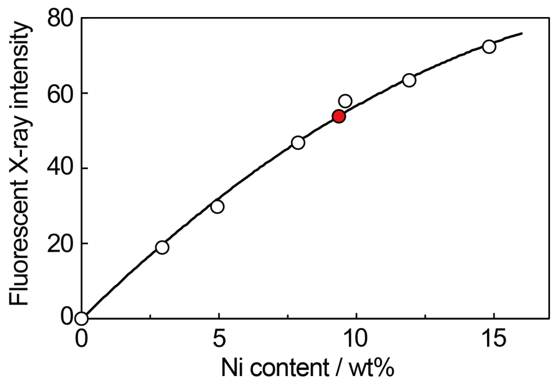

Figure S1. Calibration curve and measurement result of prepared sample in XRF analysis to analyze Ni loading.

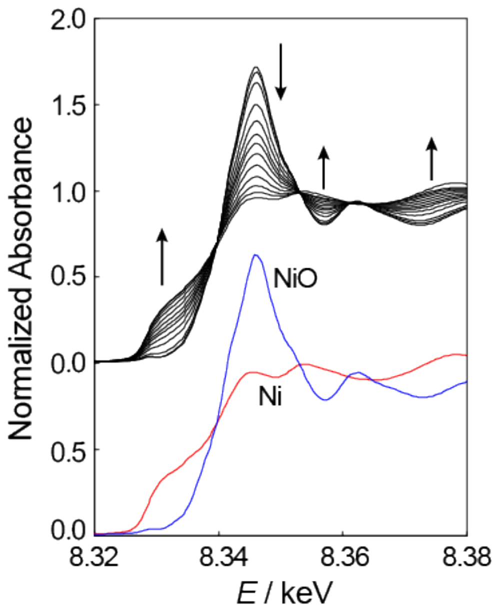

Figure S2. XANES spectral change during the TPR process for NiO particle supported on SiO_2 from room temperature to 700 °C under a diluted H_2 gas flow.

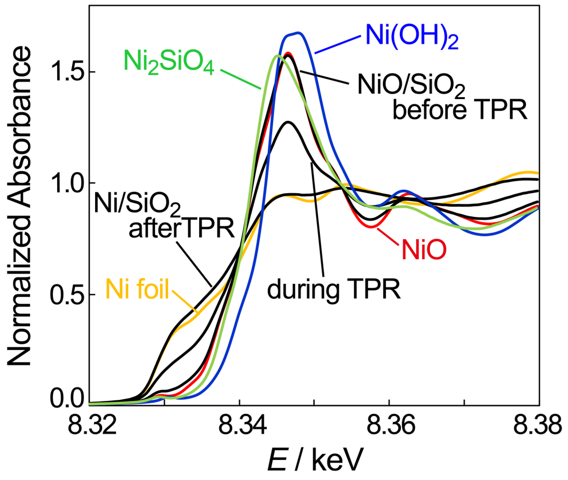

Figure S3. The XANES spectra (black line) during the TPR process of SiO_2-supported NiO particles were compared with those of NiO (red line), $Ni(OH)_2$ (blue line), Ni_2SiO_4 (green line), and Ni foil (yellow line).

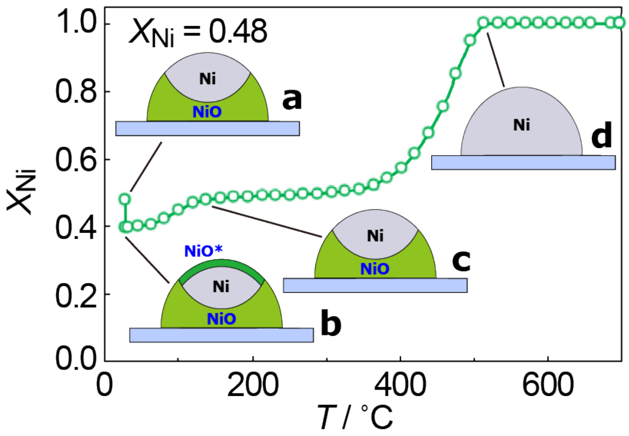

Figure S4. A schematic diagram of the series of changes in the intraparticle chemical state during this TPR process. First, NiO particles supported on SiO_2 were partially reduced via TPR until $X_{Ni} = 0.48$. This state was quenched and cooled to room temperature (**a**), and He-diluted O_2 gas was introduced to oxidize the metallic Ni site exposed on the particle surface (**b**). The NiO formed on the particle surface (labeled as NiO*) has a metallic Ni core inside, and its environment is different from that of the other NiO located at the particle surface. Subsequently, *in situ* XAFS measurements were performed on the TPR process under a flow of He-diluted H_2 gas, and it was found that reduction of an amount corresponding to NiO* had progressed at around 100 °C (**c**). Further heating led to the reduction of other NiO moieties at 450-500 °C, ultimately resulting in reduction to metallic Ni (**d**).

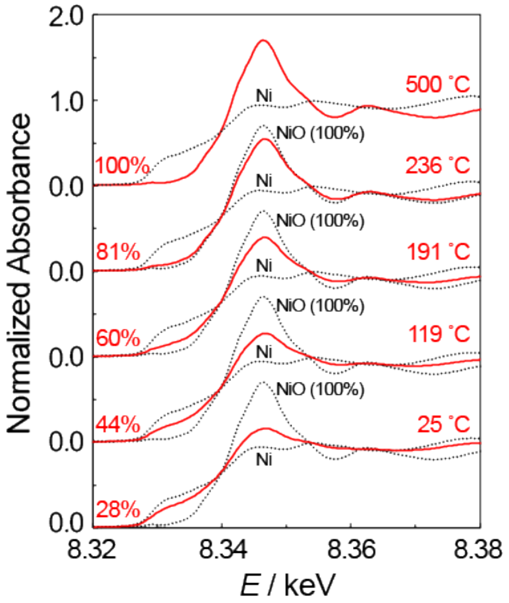

Figure S5. The XANES spectra measured for the partial oxidation treatments.

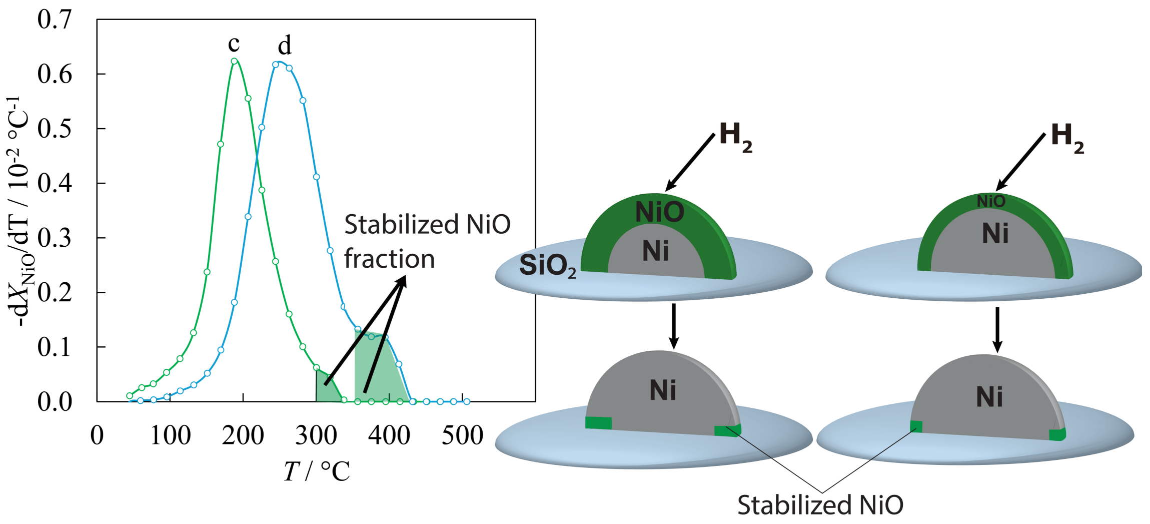

Figure S6. This diagram illustrates the behavior of the shoulder structure at high temperatures observed for the first derivative curve of X_{NiO} and the stabilization at the SiO_2–NiO interface. The partial oxidation of Ni nanoparticles forms the NiO shell on the particle surface, and the reduction of the shell by H_2 converts NiO to metallic Ni. However, because the NiO present at the interface between the particle and the support is likely stabilized via SMSI, it is expected that higher temperatures will be required for its reduction.

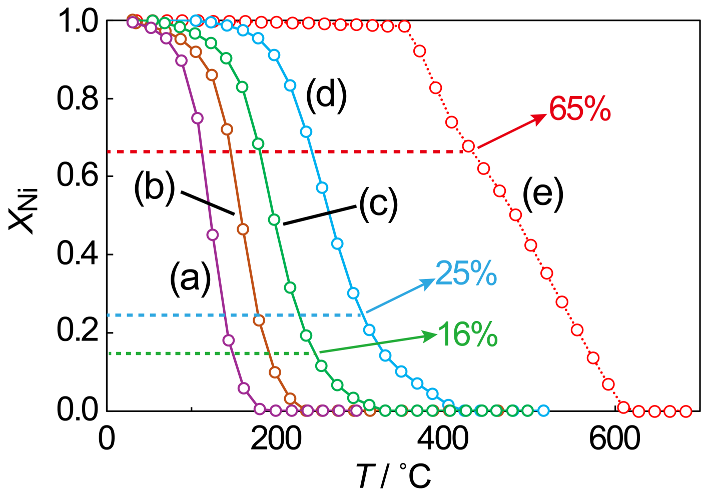

Figure S7. The data in Figure 6A are plotted normalized to a value of 1.0 for X_{NiO} at room temperature. The actual value of X_{NiO} at room temperature is 0.28 (a), 0.44 (b), 0.60 (c), 0.81 (d), and 1.00 (e).

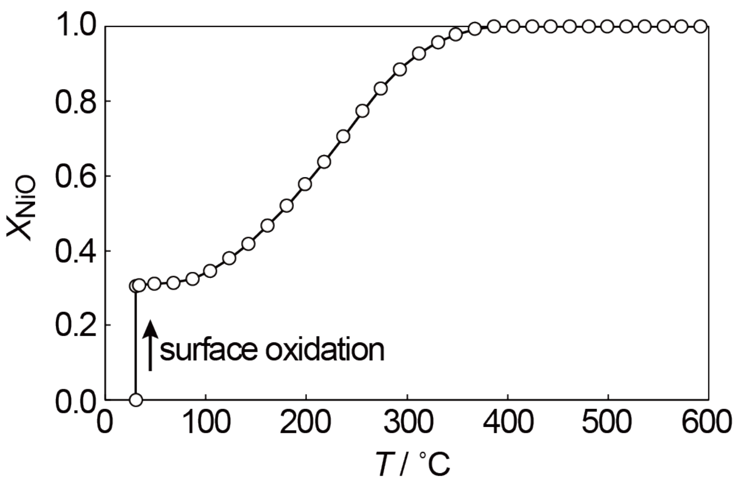

Figure S8. The temperature change of X_{NiO} during the TPO process, in which the metallic Ni particles supported on SiO_2 used in this study, with the average particle size of 6.2 nm, were directly oxidized. The 31% NiO produced at room temperature corresponds to the formation of a surface NiO layer upon exposure to O_2 gas.
